# The Accuracy of Cone Beam Computed Tomography Scans in Determining the Working Length in Teeth Requiring Non-surgical Endodontic Treatment: A Retrospective Clinical Study

**DOI:** 10.7759/cureus.59907

**Published:** 2024-05-08

**Authors:** Ayman M Abulhamael, Mohammed Barayan, Lama M Makki, Suha M Alsharyoufi, Turki H Albalawi, Shatha Zahran, Omar Alkhattab, Abrar S Kutbi, Rayan S Alrehili, Ziyad T Alzamzami

**Affiliations:** 1 Endodontics, Faculty of Dentistry, King Abdulaziz University, Jeddah, SAU; 2 Oral and Maxillofacial Radiology, Faculty of Dentistry, King Abdulaziz University, Jeddah, SAU; 3 Dentistry, King Abdulaziz University Hospital, Jeddah, SAU; 4 Dentistry, King Abdullah bin Abdulaziz University Hospital, Jeddah, SAU; 5 Dentistry, Faculty of Dentistry, King Abdulaziz University, Jeddah, SAU

**Keywords:** accuracy, cbct, non-surgical endodontic treatment, working length, apex locator

## Abstract

Background: Endodontic treatment aims to eradicate both microbial infection and inflammatory processes within the root canal space as well as in the periapical (PA) region of the tooth. To achieve this, the canals should be cleaned, shaped, disinfected, and obturated to the proper working length. Clinically, the working length is described as the measurement from the coronal reference point to the physiological apex located at the apical foramen. In the available literature, electronic apex locators (EAL) with periapical (PA) radiographs are the most reliable and precise tools for determining the working length in routine root canal treatment. Therefore, the aim of this retrospective clinical study is to evaluate if cone beam computed tomography (CBCT) scans are reliable and accurate in measuring endodontic working length compared to standard clinical measurement methods.

Methods: Patients who fit the inclusion criteria were identified. A postgraduate endodontic resident blinded to the cone beam computed tomography scan results treated all teeth in the field of view that needed endodontic treatment. The root canal length was determined using J Morita Root ZX II apex locator (J Morita Corp., Kyoto, Japan) and periapical radiographs. The dental radiology specialist interpreted the pre-existing cone beam computed tomography (CBCT) scan images and determined the working length. Statistical comparisons of the working length measurements of EAL and CBCT were performed using paired sample t-tests after verifying normality.

Results: No statistically significant differences in the working lengths were found in all canals with the exception of the palatal canal only (t=2.16, p=0.034), suggesting consistent measurements between EAL and CBCT.

Conclusion: In teeth requiring endodontic treatment, pre-existing cone beam computed tomography scan images are accurate as electronic apex locators when determining the working length. A limitation of this study is that it only includes a limited number of samples and is affected by operator variation.

## Introduction

There is no denying that dental infection is a severely painful experience that is unavoidably experienced by many people. To treat these aggressive infections and relieve their symptoms, the elimination of the bacteria is required [1]. Endodontic treatment is one of the methods that can help to treat and alleviate the pain associated with these infections [1]. The main objective of root canal therapy is to eradicate both microbial infection and inflammatory processes within the root canal space as well as in the periapical (PA) region of the tooth [2]. For this to happen, it is crucial to access, prepare, and fill the root canal system to its full working length to complete a successful root canal treatment [2]. This should be done in a way that will completely get rid of any microorganisms. As far as clinical definition is concerned, the working length is described as the measurement from the coronal reference point to the physiological apex located at the apical foramen [1]. During endodontic treatment, determining the working length of a root canal system and maintaining it for the duration of the treatment can be an extremely challenging task [3]. The accuracy rate for determining the working length in routine clinical practice varies between 55% and 93% based on the use of electronic apex locators (EAL) in conjunction with periapical radiographs, which is widely accepted as one of the most reliable and precise methods [4]. It must be noted, however, that there is a possibility that electronic apex locators may not function as intended due to several reasons that can affect the conductivity of the apex locator, such as anatomical complexities, a lack of patency, or metallic restorations [5]. The electrical conductivity of intracanal fluids such as irrigants, blood, pulp, and exudate, along with factors such as immature apex development, periradicular lesions, and apical foramen size, as well as the file size used to estimate the root canal length, may also have an impact on the accuracy of electronic apex locators [6]. Additionally, necrotic teeth, lesions on the periapical surface, or teeth with inflammatory apical root resorption may have alterations to the apical root canal structure. Thus, as a result, electronic apex locators may have difficulty detecting the apical foramen [6].

With the development of technology, cone beam computed tomography (CBCT) has gained considerable interest in the dental field since its introduction in 1998, as it can assist in diagnosing and planning treatment for a variety of conditions, including dental caries, impacted teeth, periodontal disease, and placing dental implants [7]. CBCT is an imaging system that produces images by directing a cone-shaped beam onto a two-dimensional detector, requiring only a single pass or less around the patient's head to generate a series of images. Cone beam computed tomography imaging is also used for assessing the anatomy of the jaw and the areas surrounding it [8]. In endodontics, cone beam computed tomography is becoming increasingly popular as a technique to determine the working length of teeth requiring root canal treatment because it provides a three-dimensional image of the tooth and its surroundings [8]. A cone beam computed tomography scan image could provide insights into the root canal angles, as well as identify the position of the apical foramen, which cannot be determined precisely by periapical radiographs [9]. A clearer visual representation of the root canal morphology will allow for a simpler and more precise measurement of the canal length [9]. CBCT imaging offers numerous advantages compared to traditional medical computed tomography imaging, including the smaller size, lower cost, ease of use, higher spatial resolution, image sharpness, and lower effective radiation doses, which make it a better imaging method over medical computed tomography imaging. Compared to traditional radiographic imaging, cone beam computed tomography can overcome many of the shortcomings that are associated with the apex locator and the traditional periapical radiograph [10]. The purpose of this study is to determine whether CBCT scan images accurately measure the endodontic working length and whether those measurements are comparable to those obtained by using the J Morita Root ZX II electronic apex locator (J Morita Corp., Kyoto, Japan) and periapical radiographs, which are standard clinical measurement methods.

Significance of the study

If proven that there are no differences in the measurements obtained from the cone beam computed tomography scan images and those obtained from the J Morita Root ZX II electronic apex locator and periapical radiograph, we can depend on the CBCT for working length measurement in cases where EAL cannot give an accurate reading. Also, we can guarantee the information given by the CBCT when we try to do any surgical procedure near any vital structures. Furthermore, it saves time and improves treatment quality.

## Materials and methods

This study was approved by the Research Ethics Committee of King Abdulaziz University, Jeddah, Saudi Arabia. This study is a retrospective clinical study that involves the assessment of 248 root canals of 176 patients undergoing endodontic treatment at King Abdulaziz University Dental Hospital (KAUDH), Jeddah, Saudi Arabia. Sample size calculation was performed using G*Power software version 3.1 for paired samples t-test in which the statistical power is set at 98%, with an alpha error probability of 0.01, and the effect size was estimated by considering the anticipated discrepancy between CBCT and standard measurement methods, as reported [2]. A two-tailed test was chosen to comprise potential differences in either direction. Following the calculation, it was determined that a minimum sample size of 90 teeth was necessary to ensure adequate statistical power for detecting significant disparities between the measurement techniques. Along with demographic data such as age and gender, the position of the teeth in the dental arches and the number of teeth were also recorded. It was decided to utilize a consecutive sampling method in this study to make sure that all the patients who meet the inclusion criteria will be included in the study.

To be eligible and be included in the study, the patient must be receiving dental treatment at King Abdulaziz University Dental Hospital (KAUDH), Jeddah, Saudi Arabia. Additionally, the patient must have undergone, in the past, a cone beam computed tomography scan with a limited field of view (5×5 cm) with a voxel size of 0.08 mm either for endodontic evaluation or for other reasons, as well as having teeth that need to be treated endodontically within the same field of view of the CBCT scan image. Those patients who agree to participate will be included in the study. Patients not treated at King Abdulaziz University Dental Hospital (KAUDH), Jeddah, Saudi Arabia, or those who do not have a pre-existing CBCT scan done or require endodontic treatment, and those who refuse to participate in the study are excluded. All teeth within the field of view that require endodontic treatment will be treated by a postgraduate endodontic resident blinded to the results of the CBCT scan image. The root canal length will be determined using J Morita Root ZX II apex locator and periapical radiographs.

In this retrospective study, CBCT data was obtained using KaVo OP3D Pro (KaVo Kerr, Brea, CA) limited FOV CBCT scan (5×5 cm) with a voxel size of 0.08 mm. The CBCT exposure parameters were 8.2 mA and 90 kV, with a scanning time of six seconds. The images were presented on a calibrated Dell monitor, featuring a 17-inch display with a dot pitch of 0.28, capable of high resolution. The monitor was set to a color depth of 16 bits and a screen resolution of 1,024x768 pixels. Identification of teeth requiring endodontic treatment was performed through the limited field of view in the CBCT scan images. The J Morita Root ZX II apex locator, alongside periapical radiographs, was used by the postgraduate endodontic residents to measure the working lengths of the canals with the standard of 0.5 mm from the apex, which were then recorded in their progress notes.

Then, measurements were collected from both the working lengths noted by the postgraduate endodontic residents using the apex locator and the working lengths of the root canals on the CBCT scan images. Afterward, both measurements were uploaded into the same spreadsheet for statistical analysis. The electronic apex locator readings of all patients were collected by one researcher, while another researcher collected the working length measurements from the cone beam computed tomography scan images. To avoid conflict of interest, the researchers did not disclose their readings to each other as a precaution, which ensured inter-examiner agreement.

**Figure 1:**
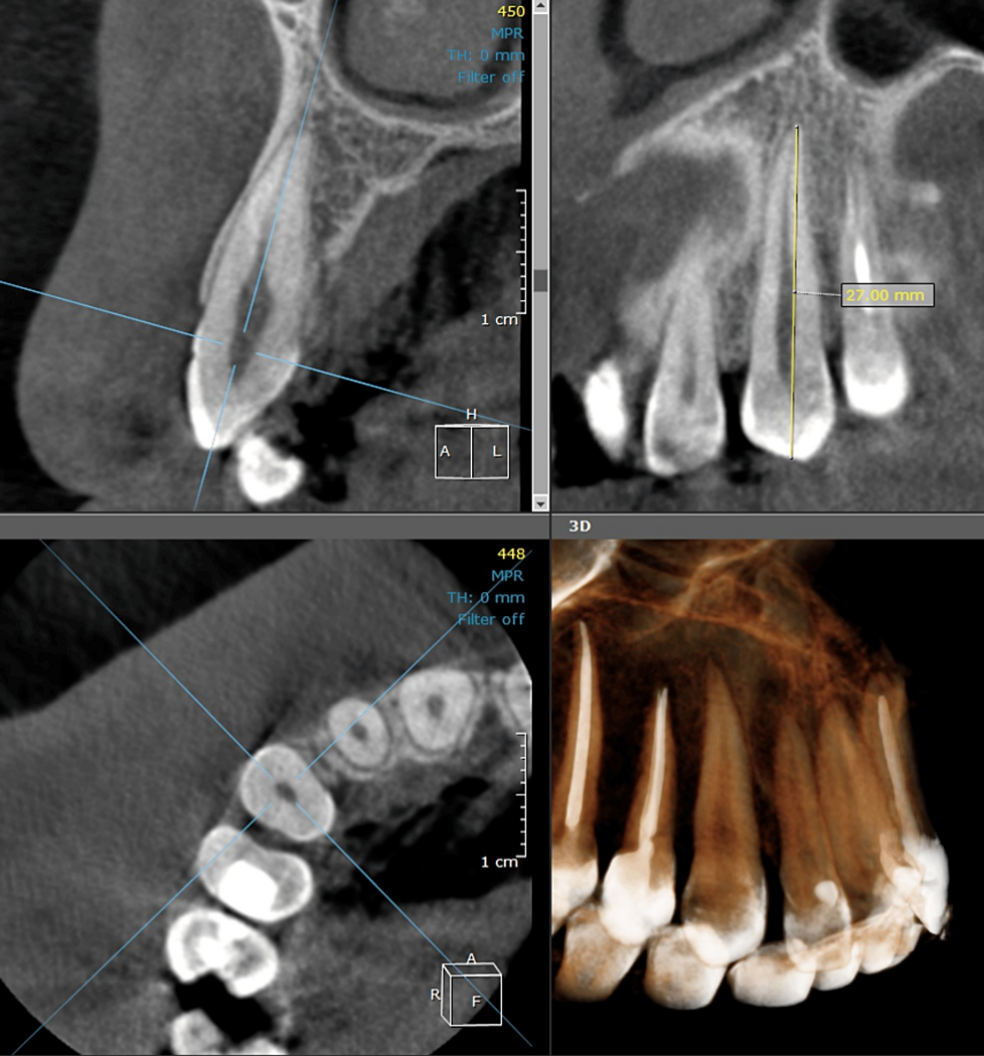
Measuring the root length in the corrected sagittal view

Two examiners reviewed the CBCT scans using OnDemand software (Cybermed Inc., Daejeon, South Korea). The images were examined in a dimly illuminated room devoid of windows. Following adjustments to image contrast and utilization of the magnification tool for enhanced visualization, the selected sections were parallel to the long axis of the root tips in the corrected sagittal view, and root canal length was determined (Figure 1).

After two weeks, CBCT images were re-evaluated independently by the same observers to measure intra-observer agreement. Measurements were then collected on a single Excel spreadsheet (Microsoft Corp., Redmond, WA) and analyzed with the help of a biostatistician, and the differences between the two methods were calculated using a t-test.

## Results

This study included 176 teeth, comprising 248 root canals. All subjects received treatment at King Abdulaziz University Dental Hospital (KAUDH), with CBCT scans acquired before any endodontic treatment. The sample comprised 56.8% females and 43.2% males, and the majority of teeth were posterior. Descriptive statistics were used to summarize various study characteristics, including gender, tooth type, number of roots per tooth, and number of canals per tooth. Comprehensive details regarding the included teeth are presented in Table 1. Moreover, mean values and standard deviations were calculated for both EAL and CBCT measurements and reported in millimeters. Statistical analysis was conducted using the paired sample t-test to compare the mean working lengths of root canals paired observations measured by EAL and CBCT. Prior to analysis, normality assumptions were assessed using the Shapiro-Wilk test and visual inspection of histograms. All statistical analyses were carried out using Statistical Package for the Social Sciences (SPSS) version 28 (IBM SPSS Statistics, Armonk, NY), with significance level set at α=0.05.

**Table 1:** Description of the included teeth (N=176)

	Number	Percentage
Gender		
Female	100	56.8
Male	76	43.2
Tooth type		
Anterior	42	23.9
Posterior	134	76.1
Number of roots per tooth		
Single rooted	70	39.88
Two roots	60	34.1
Three or more	46	26.1
Number of canals per tooth		
Single canal	69	39.2
Two canals	27	15
Three canals	64	36.4
Four canals	15	8.5
Five canals	1	0.6

The results of the paired sample t-test revealed a statistically significant difference between the mean working lengths determined by the two methods in the palatal canal only (t=2.16, p=0.034). Specifically, the mean working length of palatal canals measured by EAL was found to be 19.2 mm (±0.28), whereas the mean working length of palatal canals obtained through CBCT was slightly greater with a mean of 19.4 mm (±0.29). However, for all other canals, no significant differences were found in the working length measurements obtained by the EAL and CBCT methods, indicating an overall consistency between these two approaches as detailed in Table 2.

**Table 2:** Analysis of mean working length (mm) comparisons between CBCT and EAL methods, as determined by the dependent t-test, showing the mean (±standard deviation) values in millimeters *Statistically significant WL: working length, EAL: electronic apex locator, PA: periapical radiograph, CBCT: cone beam computed tomography

Root canal ID	Number of measured canals	Mean WL (EAL and PA radiograph)	Mean WL (CBCT)	Mean difference (EAL-CBCT)	P values
Buccal	85	21.06 (±0.24)	21.2 (±0.24)	-0.13 (±0.07)	0.058
Palatal	65	19.2 (±0.28)	19.4 (±0.29)	-0.16 (±0.07)	0.034*
Mesial	3	20.33 (±1.2)	20.44 (±1.49)	-0.4 (±0.28)	0.73
Distal	39	19.1 (±0.32)	19.06 (±0.30)	0.04 (±0.09)	0.68
Mesiobuccal	26	19.08 (±0.36)	19.05 (±0.38)	0.03 (±0.14)	0.835
Mesiolingual	13	18.84 (±0.6)	18.99 (±0.55)	-0.15 (±0.13)	0.28
Distobuccal	17	17.94 (±0.42)	16.9 (±1.13)	1.03 (±1.01)	0.32

## Discussion

To achieve the best possible results from a root canal treatment, it is important to determine the working length accurately [6]. Various methods are employed for the determination of the working length, including periapical radiographs and electronic apex locators. Additionally, there are other techniques that can provide additional information, including digital tactile sense, apical periodontal sensitivity, and paper cones [8]. It is not possible to rely on tactile perception and apical periodontal sensitivity. Additionally, it is not possible to determine the working length with precision using paper cones because of their piston-like effect. In endodontics, periapical radiographs estimate a root canal length; however, they have several drawbacks. An important point to keep in mind is that periapical radiographs only show a two-dimensional image of the three-dimensional object. In addition, overlapping anatomical structures often prevent the apex of the root from being seen on a radiograph. It is also possible that periapical radiography can result in distortion of images from time to time. Moreover, the radiographic working length is often overestimated as a consequence of this method of radiography [10].

It is recommended that instrumentation should not extend beyond the apical foramen and should be limited to a distance of 0-2 mm shorter than it. Since the apical foramen cannot be detected on two-dimensional periapical radiographs, usually, the radiographic apex must be used as an apical reference point. Up to 92% of teeth have an apical foramen that deviates from the radiographic apex of the root in all areas, and the foramen may be as much as 3.8 mm shorter than the radiographic apex. Several studies have demonstrated that the radiographic apex is not an accurate measurement of the apical extent of root fillings. An electronic apex locator is typically used along with a periapical radiograph to determine the length of a root canal [5]. To determine root canal working length, clinical practitioners generally rely on electronic apex locators, as they are accurate and reliable. Although electronic apex locators are generally reliable, there is the possibility that they may not perform as expected [11].

In contrast to traditional radiographic imaging modalities, cone beam computed tomography overcomes many of the shortcomings of those modalities [8]. Endodontists have benefited greatly from cone beam computed tomography scanning in recent years as part of their research and clinical practice. Cone beam computed tomography imaging should, however, be evaluated critically in each case. It is also necessary to process previously performed images in each perspective so that the most information can be derived from the volume and to meet the standards of the as low as reasonably achievable (ALARA) principle [3]. Our area of interest and its surrounding structures can be viewed in three dimensions using the cone beam computed tomography scanner, as it allows us to view images in three planes simultaneously: mesiodistal, buccolingual, and coronal. Additionally, it is possible to determine accurately the working length of the root canals with cone beam computed tomography [12]. It has also shown that cone beam computed tomography scan images give more reliable results than electronic apex locator measurements [13,14]. To improve the accuracy of working length measurements, a better visual representation of root canal morphology is necessary [6]. In addition to providing a better understanding of tooth anatomy, cone beam computed tomography could also be used to diagnose periapical pathologies, root fractures, and internal and external root resorptions [4]. It should be possible to improve the accuracy of working length measurement by improving our understanding of the three dimensions of dental roots. This means that by measuring root canal lengths with cone beam computed tomography scans in advance of endodontic treatment, one can optimize treatment results. Using this method may be an effective way to determine how long the root canals are before they are treated by an endodontist and plan for treatment accordingly [2].

It was essential to include all patients who met our inclusion criteria to be able to achieve the most successful results. As a precautionary measure to ensure that the results of the cone beam computed tomography scan will not influence the measurements obtained by the endodontic residents when treating patients with electronic apex locators and periapical radiographs, the treating postgraduate endodontic residents were blinded to these results.

It was found that there was no significant difference between the measurements obtained by cone beam computed tomography scans and those obtained by electronic apex locators when comparing the results of both methods, except for the palatal root, which makes the CBCT scan more superior to the PA radiographs in tracking the end of the root especially that usually this root is overlapping with many anatomical structures in the area. Therefore, if cone beam computed tomography scan images are available, it will be possible to calculate the root canal system's working length before the patient visits the endodontist and the treatment can then be planned accordingly.

Similar clinical studies were conducted by Jeger et al. (2012) [15] and de Morais et al. (2016) [2], and they found that limited CBCT scans can be used for endodontic working length measurements. Our result goes along with their conclusion, that cone beam computed tomography can provide useful information regarding the root canal system and roots, as well as determine root canal lengths, and should be used whenever possible. Additionally, in studies done on extracted teeth by Liang et al. (2013) [14], Ghule and Naik (2019) [11], Mahmoud et al. (2021) [1], and Yılmaz et al. (2017) [4], they all agreed that cone beam computed tomography offers greater precision and potential than electronic apex locators when it comes to determining the working length of the root canal system. However, in studies conducted by Lucena et al. (2019) [10] and Pham and Khuc (2020) [8], their results demonstrated that electronic apex locators were more accurate when determining the root canal working length.

It is advantageous to use cone beam computed tomography rather than electronic apex locators or periapical radiographs, since it provides us with a three-dimensional understanding of the root canal system and its surrounding structures, unlike periapical radiographs, which provide only a two-dimensional image [2]. Additionally, cone beam computed tomography assists us in planning and reducing treatment time [1,16].

A substantial amount of further research is, however, required, since most of the studies conducted to date have been carried out on extracted human teeth without taking the oral environment into account, as well as other external factors.

Limitations of the study

The limitations of this study is that it only includes a limited number of samples and is affected by operator variation.

Impact of the study

It is important to note that this study will have an impact on both patients and endodontists, as advanced proper planning of cone beam computed tomography measurements will help reduce the length of time required for treatment.

## Conclusions

In conclusion, in teeth requiring endodontic treatment, pre-existing cone beam computed tomography scan images are accurate as electronic apex locators when determining the working length. Furthermore, when dealing with maxillary posterior teeth where anatomical structures can make it difficult to locate the apex of the tooth, CBCT can provide accurate location and measurements of the palatal root compared to the two-dimensional image after using EAL, which is anatomically challenging to track, particularly in periapical radiographs.
